# A Case Report of a Rare Mesenteric Schwannoma: A Diagnostic Challenge

**DOI:** 10.7759/cureus.71206

**Published:** 2024-10-10

**Authors:** Samrat Shrestha, Mandesh Shrestha, Mecklina Shrestha, Bijay Raj Bhatta

**Affiliations:** 1 Department of General Surgery, Bir Hospital, National Academy of Medical Sciences (NAMS), Kathmandu, NPL; 2 Department of Surgical Gastroenterology, Bir Hospital, National Academy of Medical Sciences (NAMS), Kathmandu, NPL; 3 Department of Emergency Medicine, Manmohan Memorial Medical College and Teaching Hospital, Kathmandu, NPL

**Keywords:** case report, immunohistochemistry, laparotomy, mesentery, s-100, schwannoma

## Abstract

Schwannomas, also known as neurilemmomas, are neurogenic, benign tumors of Schwann cells arising from peripheral nerve sheaths that may be present at almost any anatomical site. A primary mesenteric schwannoma is an extremely rare tumor, with only 12 cases reported in the literature to date. Preoperative diagnosis of mesenteric schwannomas is almost impossible because of the rarity of the case and their nonspecific symptoms. Instead, we made the diagnosis postoperatively using histopathological examination (HPE) and immunohistochemistry (IHC) following the complete surgical resection of the mass. We present the case of a 39-year-old female with painless, progressively increasing abdominal mass for two years. Her preoperative clinical diagnosis was inconclusive, and a diagnosis of a primary mesenteric schwannoma was made after HPE and IHC reports following en bloc resection of the mass.

## Introduction

Schwannomas are slow-growing benign mesenchymal neoplasms of the peripheral nerve sheath, which are usually present in young to middle-aged individuals with equal sexual predilection. The pathogenesis of schwannomas is the formation of defective Merlin protein due to inactivating germline mutation in tumor suppressor gene NF2 on chromosome 22. They are commonly located in soft tissues of the head and neck, flexor surface of extremities, vestibular branch of the 8th cranial nerve, and rarely in the mediastinum, retroperitoneum, visceral organ, and gastrointestinal tract [1,2]. Of all gastrointestinal tract submucosal tumors, gastrointestinal schwannomas account for 0.4% to 1% of cases, most commonly arising from the stomach [3], whereas primary mesenteric schwannomas are very rare. Secondary degenerative changes such as hyalinization, hemorrhage, calcification, and cyst formation are occasionally seen in schwannomas [2]. A complete surgical excision with a clear margin is the mainstay of treatment, while recurrence or malignant transformation following surgery is extremely rare [1-3]. We are presenting a 39-year-old female with a slow-growing abdominal mass to consider a mesenteric schwannoma as a differential diagnosis in inconclusive cases.

## Case presentation

**Figure 1:**
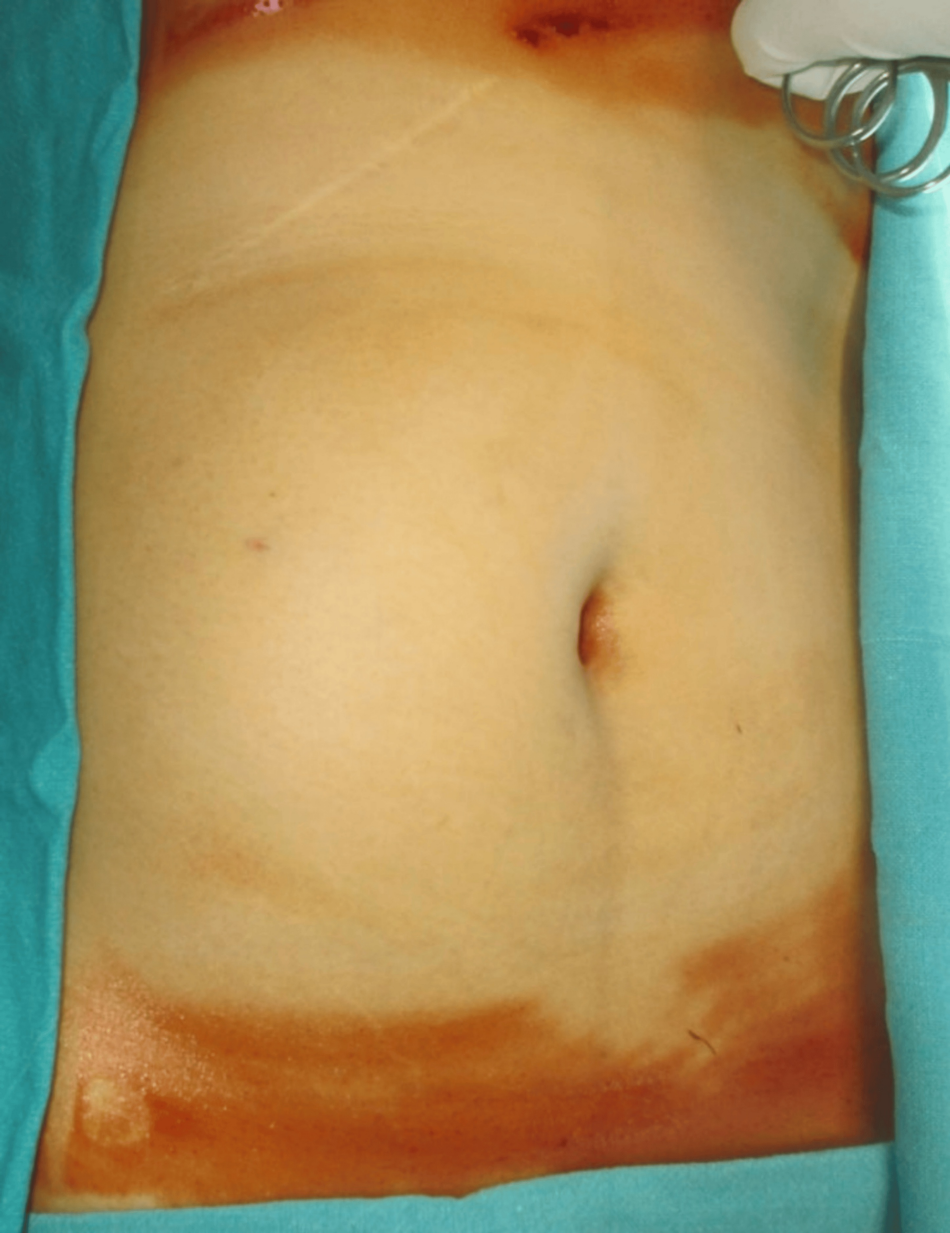
Preoperative image of abdominal mass

A 39-year-old female presented to the outpatient department (OPD) of our hospital with a history of painless, progressively increasing abdominal mass over the past two years. She had undergone open cholecystectomy for symptomatic cholelithiasis seven years ago. On physical examination, the abdomen was distended with an approximately 15*10 cm palpable, smooth, well-defined, mobile mass extending from the right hypochondrium to the right lumbar region (Figure 1). All other blood investigations, including tumor markers, were unremarkable.

**Figure 2:**
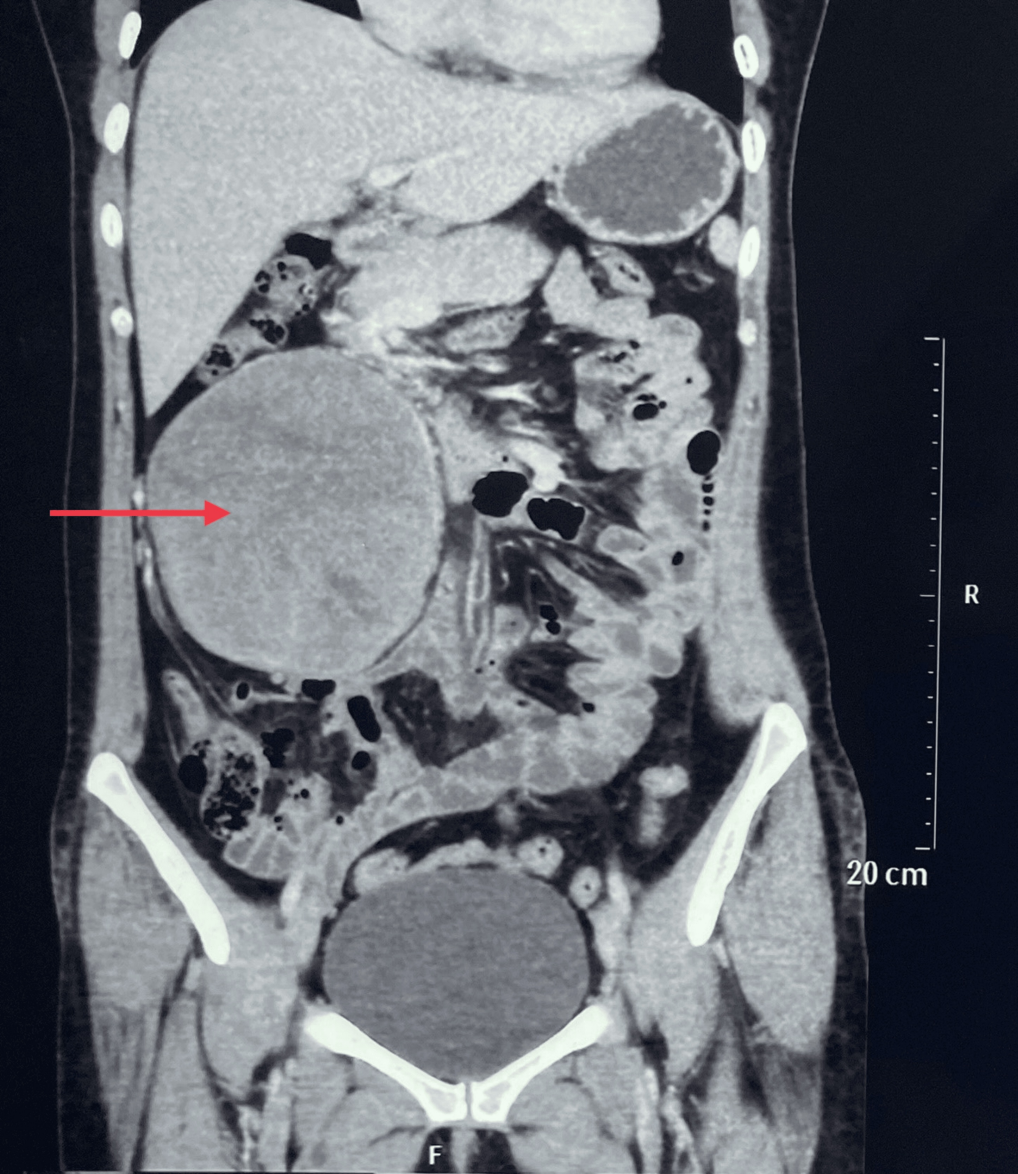
CECT abdomen coronal section showing a heterogeneously enhanced well-defined soft tissue density mass in the right hypochondriac and lumbar region (red arrow). CECT: Contrast-Enhanced Computed Tomography

**Figure 3:**
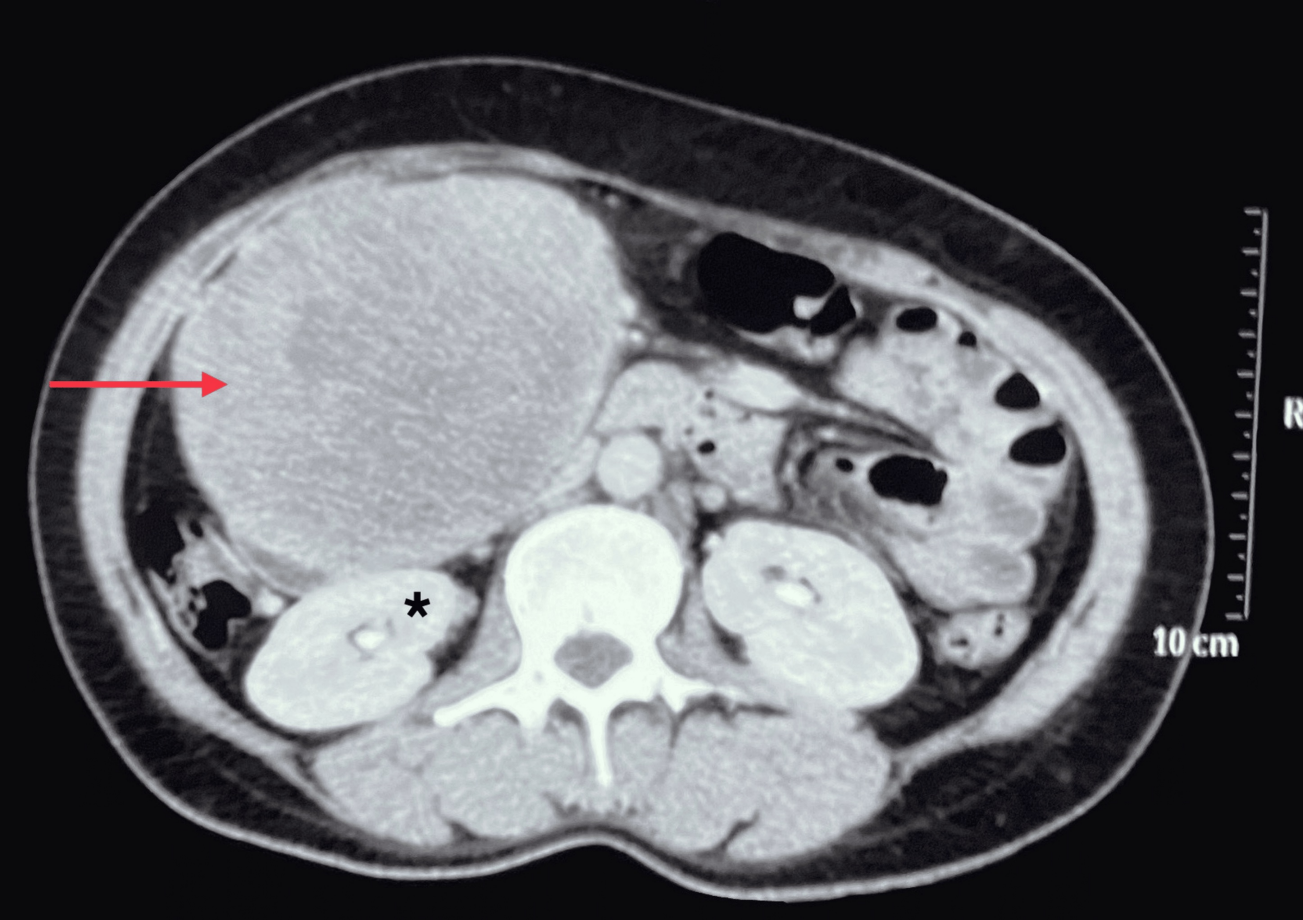
CECT abdomen axial section showing a heterogeneous mass abutting the anterior abdominal wall anteriorly and right kidney posteriorly (asterisk) CECT: Contrast-Enhanced Computed Tomography

Ultrasonography (USG) revealed a well-defined isoechoic mass lesion measuring approximately 10.9*9.9 cm in the right quadrant of the abdomen. Contrast-enhanced computed tomography (CECT) showed a well-defined soft tissue density mass measuring approximately 12*11*10 cm in the right abdominal cavity involving the right hypochondrium and lumbar region arising from the second part of the duodenum (Figure 2). The mass abutted anteriorly to the anterior abdominal wall and posteriorly to the second and third parts of the duodenum, the lower part of the right kidney, and medially to the inferior vena cava (IVC) (Figure 3). Blood supply to the mass was from the superior mesenteric artery and drained to the superior mesenteric vein. 

**Figure 4:**
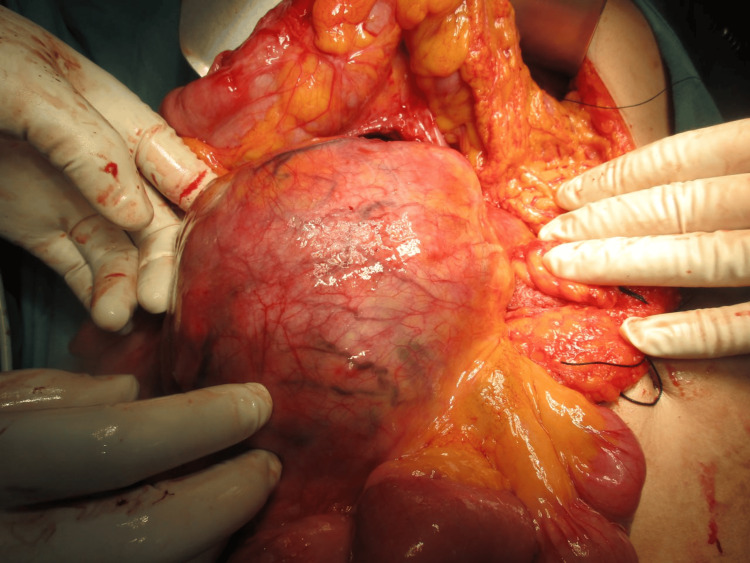
Intraoperative image of the mass located in mesentery of small bowel and proximal colon

**Figure 5:**
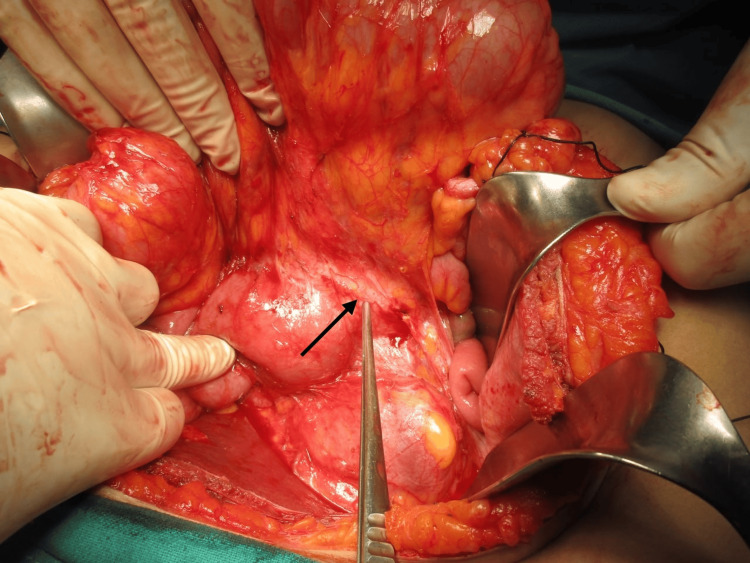
Tumor lifted and arrowhead showing superior mesenteric vessels

**Figure 6:**
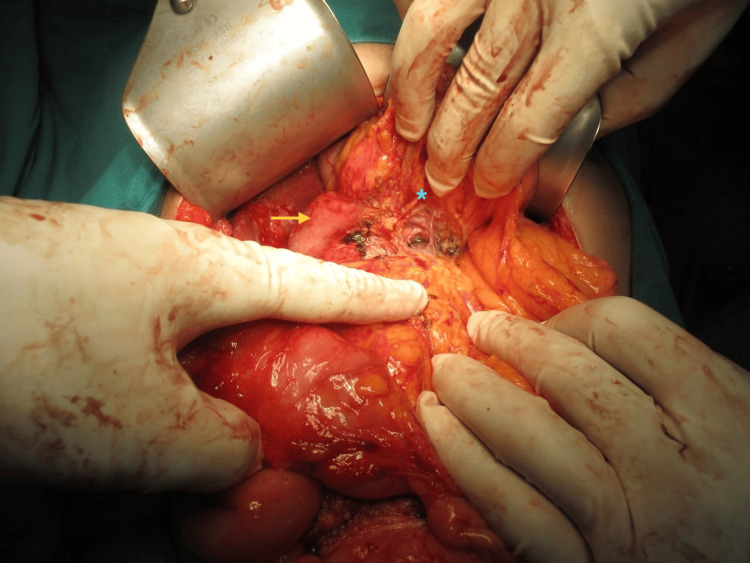
Tumor separated from the pancreatic head (asterisk) and uncinate process. The yellow arrow shows the duodenum

An exploratory laparotomy was scheduled after a preoperative diagnosis of a duodenal gastrointestinal stromal tumor was established. Following Chevron incision, Cattell Braasch maneuver and extended Kocherization were done, and the mass was freed from the third and fourth parts of the duodenum. Approximately 12*12*10 cm soft to firm spherical, well-encapsulated mobile mass was present in the mesentery of the distal small bowel and proximal colon (Figure 4), abutting posteriorly the right kidney and renal vein, medially to the IVC, 3rd and 4th parts of the duodenum, head, and uncinate process of the pancreas. Blood supply of the mass was from superior mesenteric vessels (Figure 5). The mass was dissected and released from its attachment (Figure 6), and excised en bloc from the mesentery. Ileocecal resection was done after inspecting the viability of the distal ileum and proximal colon, followed by side-to-side ileocolic stapled anastomosis. The patient’s overall postoperative course was uneventful and discharged on the sixth postoperative day.

**Figure 7:**
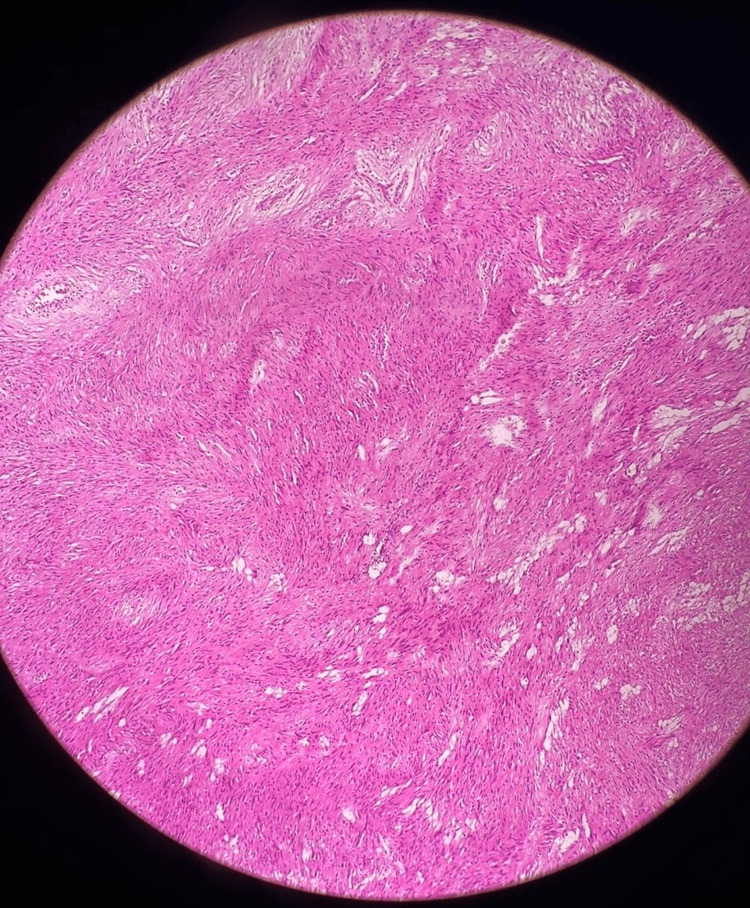
HPE of the tumor shows biphasic hypercellular and hypocellular areas on H and E stains on 10x magnification HPE: Histopathological Examination; H and E: Hematoxylin and Eosin

**Figure 8:**
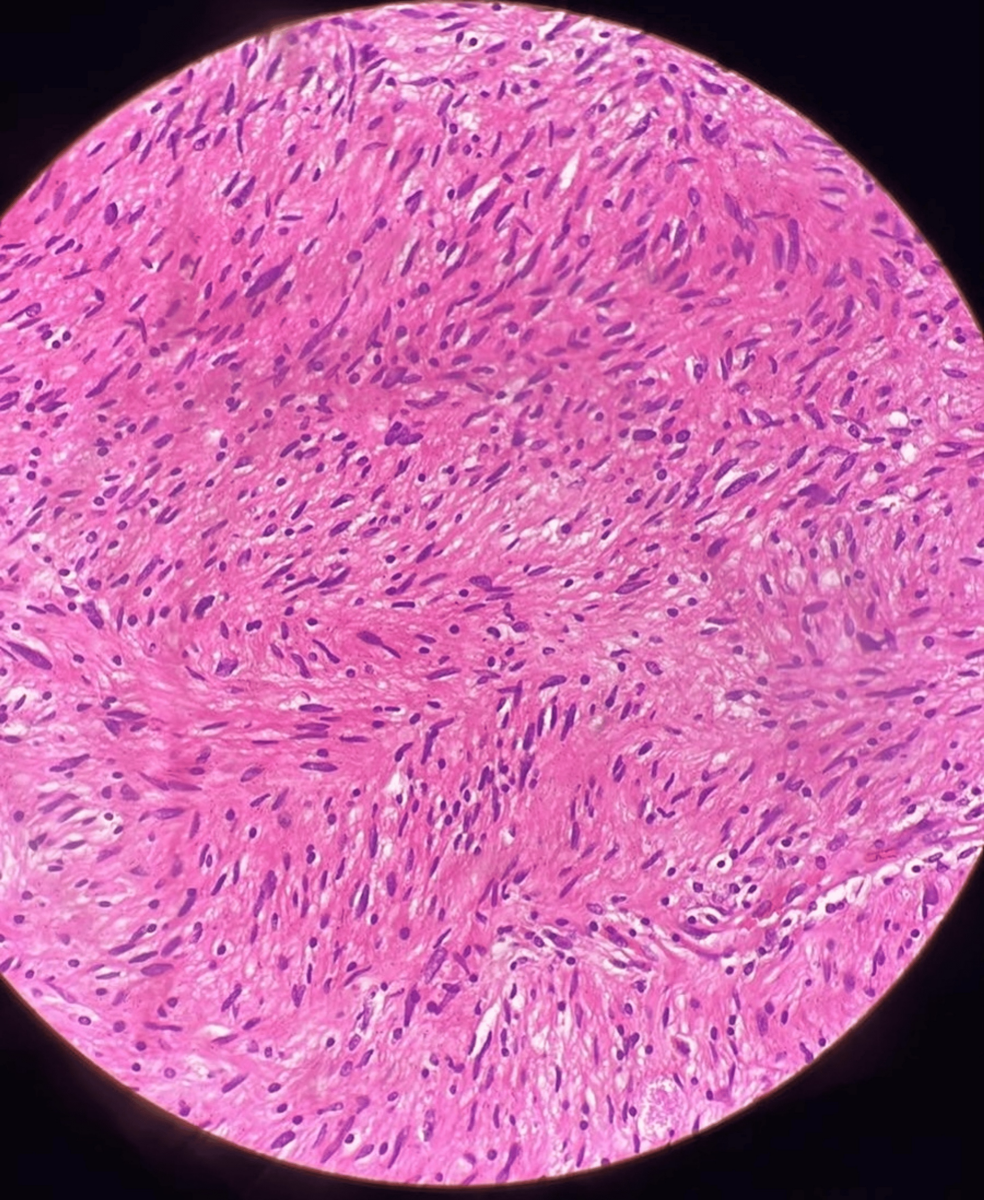
HPE of the tumor shows biphasic hypercellular and hypocellular areas on H and E stains on 40X magnification HPE: Histopathological Examination; H and E: Hematoxylin and Eosin

Histopathological examination revealed capsulated tissue with the biphasic hypercellular area (Antoni A type) with nuclear palisading and central nuclear-free zones (Verocay bodies) and hypocellular (Antoni B) areas (Figure 7 and Figure 8). The tumor was positive for immunohistochemical staining of S100, vimentin, and SOC-10 and negative for smooth muscle actin, Melan-A, CD34, Desmin, Calretinin, CD117, and CD56. The cell proliferation index, measured with Ki67 staining, was 2-3%.

**Figure 9:**
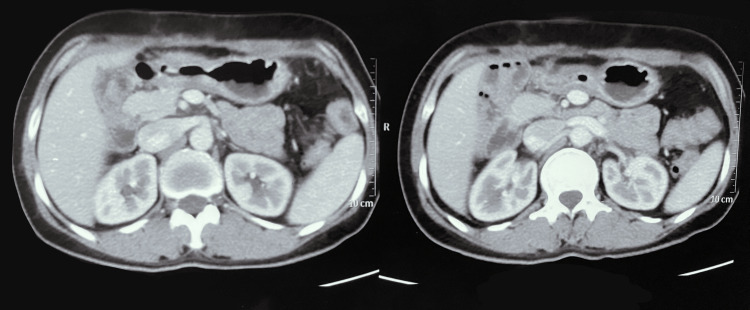
Follow-up CECT abdomen and pelvis shows no evidence of recurrence CECT: Contrast-Enhanced Computed Tomography

During one-month and six-month follow-up in the outpatient department, the patient did not have any complications and no evidence of recurrence as confirmed by a CECT abdomen and pelvis scan (Figure 9).

## Discussion

Schwannomas are usually benign (>90%), slow-growing, encapsulated tumors of peripheral nerve sheath of mesenchymal origin. Most of the cases are sporadic, and NF2, schwannomatosis, and Carneys complex have been linked with schwannoma [2-5]. The exact pathogenesis of schwannomas has not been established, but recent literature indicates a defect in merlin/schwannomin (NF2 gene product) linked to both sporadic and familial cases of schwannomas [5]. Schwannomas are tumors of young to middle age with equal sexual predilection, commonly seen in the soft tissue of the head, neck, and extremities. At the same time, other locations such as the mediastinum, retroperitoneal space, and gastrointestinal tract are uncommon, and primary mesenteric schwannomas are exceedingly rare [2,4,6]. 2-6% of gastrointestinal mesenchymal tumors are schwannomas; the most common site being the stomach (60-70%), followed by the colon and rectum [3]. Malignant transformation of schwannomas is a rare event and, if present, is associated with poor prognosis [5]. Simon GC in 1969 documented the first case of mesenteric schwannoma [1,6]. To date, only 12 mesenteric schwannomas have been reported in the literature [4,7,8]. 

Clinically, primary mesenteric schwannomas are asymptomatic, with nonspecific gastrointestinal symptoms and incidentally detected; thus, a definitive preoperative diagnosis is almost impossible [1,2]. However, these tumors may present acutely as intestinal obstruction secondary to mass effect [6]. In our case, the patient presented with an asymptomatic, palpable abdominal mass.

On USG, mesenteric schwannoma appears as a well-defined hypodense lesion. CT and magnetic resonance imaging (MRI) findings are nonspecific. Noncontrast CT shows well-circumscribed homogeneous densities. On CECT, pathological Antoni A areas are well enhanced due to hypercellularity, and the Antoni B area, composed of loose stroma and low cellularity, appears hypodense, giving an overall heterogeneous appearance [2,9]. Heterogeneity also indicates secondary degenerative changes such as cystic degeneration, hemorrhagic, or xanthomatous change [1]. T1-weighted MRI demonstrates low-intensity signals, while T2-weighted images show masses of high-intensity signals [9]. These imaging modalities are useful tools for establishing the diagnosis of primary mesenteric schwannomas; however, it is challenging to acquire an accurate preoperative diagnosis of mesenteric schwannomas. Nonetheless, these imaging techniques are useful to determine the size, location, and extent of these tumors [2,6].

The biphasic pattern: Antoni A and Antoni B is the histological hallmark that distinguishes schwannoma. The Antoni A area is a compact zone characterized by hypercellularity and long spindle cells creating a palisading pattern (Verocay bodies). The Antoni B pattern consists of a hypocellular zone of loosely arranged cells with macrophage infiltrate, often showing myxoid or hyaline degeneration [10,11]. In a systematic literature review by Bohlok et al., the biphasic histological pattern was seen in 19.2%, Antoni A in 57.7%, and Antoni B in 23.1% [12]. Malignant change in schwannomas is extremely rare and manifests as epithelioid malignant peripheral nerve sheath tumor, angiosarcoma, or round cell malignancy [11]. 

Immunohistochemistry diagnosis of schwannomas is considered the gold standard along with HPE. Schwannomas show strong immunoreactivity for S-100 (97.9%) and occasionally stains for vimentin (13.5%), while other markers like Desmin, CD-34, DOG-1, CD117 (c-KIT), and smooth muscle antigen (SMA) are negative [12,13]. Ki-67 indicates the malignant potential of the tumor, with Ki-67 >5% indicating an aggressive tumor and >20% highly predictive of malignant potential [3,4,12,13]. In our case, the Ki-67 index was 2-3%. However, the malignant potential of mesenteric schwannomas also depends on the tumor size, mitotic index, MIB-1, and local or distant metastasis [12].

Surgical excision with clear margins is the mainstay of treatment for mesenteric schwannomas with a good prognosis and the recurrence rate is very low [1,2,6]. The majority of mesenteric schwannoma case reports have not reported any indication of recurrence throughout the follow-up period [2,4,6,9]. In a literature review by Hong et al., 105 patients with gastric schwannomas, whose median follow-up time ranged from 22 to 132 months, concluded that there had been no recurrence in the follow-up time [14]. Primary mesenteric schwannomas are benign tumors, and malignant transformation is a rare event [1,2,4,6]. There is no documented case report of the malignant transformation of mesenteric schwannomas in the literature; nevertheless, case reports regarding the malignant transformation of gastric schwannomas exist [15]. In our case, after six months of postoperative follow-up, the patient has no evidence of recurrence or malignant transformation.

## Conclusions

Primary mesenteric schwannomas are extremely rare with only 12 cases reported to date. The clinical presentation of these tumors tends to be nonspecific, with some being asymptomatic incidental findings and a few being slow-growing palpable masses. They are often difficult to diagnose clinically or radiologically and always require HPE and IHC to establish a definitive diagnosis. Complete surgical excision is usually curative, with recurrence and malignant transformation extremely uncommon. Mesenteric schwannomas should be taken into consideration as a differential for slow-growing, painless abdominal mass to lessen the diagnostic burden.
